# Low Voltage Electric Current Causing Ileal Perforation: A Rare Injury

**Published:** 2016-04-24

**Authors:** Aditya Pratap Singh, Vinay Mathur, Ramesh Tanger, Arun Kumar Gupta

**Affiliations:** Department of Pediatric Surgery, SMS Medical College Jaipur, Rajasthan, India

**Keywords:** Electric burn, Perforation, Peritonitis, Children

## Abstract

Post-electric burn ileal perforation is a rare but severe complication leading to high morbidity and mortality if there is delay in diagnosis and management. We are describing a case of electric current injury of left forearm, chest, and abdominal wall with perforation of ileum in an 8-year old boy. Patient was successfully managed by primary closure of the ileal perforation.

## INTRODUCTION

Electric injuries are of two types; low tension and high tension injuries with 1000 volt as a dividing line between them.[1] Electrical injury is produced by the conversion of electric energy into heat while passing through tissues.[2] Clinical manifestation can range from no apparent injuries to serious systemic damage. Abdominal visceral injuries are associated with high voltage injuries where the contact point is directly over the abdomen.[3] There can be direct consequences of the passage of electrical current through the abdominal viscera or a complication of electric injury such as curling ulcer. These intra-abdominal electric injuries are difficult to diagnose. Abdominal visceral injuries associated with low voltage current are a rare.[4]

## CASE REPORT

An 8-year old male child presented after 10 days of sustaining electric burn over left forearm, chest, and abdominal wall. He sustained electrical burn accidently after touching electrical cable which was described as low voltage current. After sustaining electric burn, patient was brought to the private hospital. They managed patient with dressing and medications. There were no abdominal complaints at that time so he was discharged. After a week he developed abdominal pain with distension. On examination, there was necrosis of skin with eschar on the left lower abdomen with entry wound on left forearm and exit wound over left lower abdomen. He was dehydrated with pulse 100/mint. It was 2nd degree burn. Abdomen was distended and tender. On investigation hemoglobin was 11.7 gm%, TLC- 17000/mm3, serum electrolytes, liver function test, serum amylase and lipase were in normal limits. In urine analysis albumin was present. ECG was normal. Ultrasound abdomen showed thick inter-loop collection, dilatation of small bowel loops, and collapsed colon. X-ray abdomen revealed free gas under both domes of diaphragm. He was diagnosed as having bowel perforation leading to peritonitis. Immediate exploratory laparotomy was performed. There was around 50 cc of bilious fluid in peritoneal cavity with plenty of flakes over intestine. On further exploration, a perforation (0.5x0.5cm) found in the proximal ileum. Refreshing of the margins of perforation was done and primary closure performed (Fig. 1). Patient recovered uneventfully and was discharged on 7th postoperative day. Electric burn healed at the time of discharge. At follow up patient remained healthy.

**Figure:**
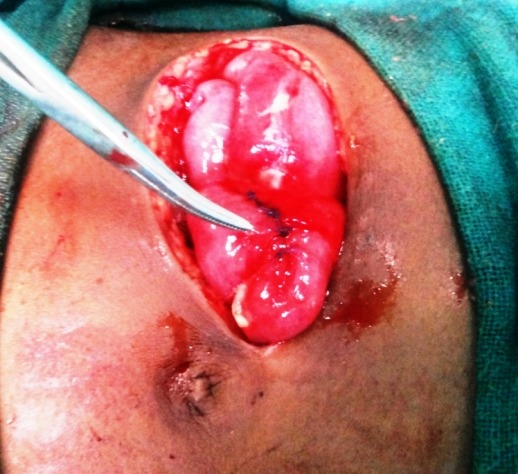
Figure 1a: Peroperative photo after perforation repair.

## DISCUSSION

Electrical burns account for approximately 5% of the hospital admissions.[5] During electrical injury, current is passed through underlying structures and cause coagulative necrosis. During passage of current passes through tissues, heat is generated according to Joule’s law (heat = I2 current × R resistance). The increase in temperature causes the denaturation of the macro molecules which is usually irreversible.[6] The resistance of the tissues during the passage of an electrical current is variable (lower for nerves and vessels and higher for fat and bones). The trauma as a result of an electrical current varies according to the individual susceptibility and the quality of care provided at the site of the accident.[7]

Few cases of entero-cutaneous fistulae and bowel perforation following electric current are reported in literature.[3,4,8]. Abdominal visceral perforation seems to be a direct consequence of the electric current rather than a complication of the injury, and often associated with high voltage current. Abdominal visceral injury is rarely associated with low voltage current, perhaps because the current density and hence heat dissipated are comparatively less. In our case the injury was due to a low voltage current. The part of bowel injured was near to the exit wound. A very small area might have affected by the electric current which would have given way after a week as our patient remained asymptomatic for more than a week.

In visceral injuries the colon and small intestine are the organs most frequently affected. Depending upon the extent of the perforation, anatomical site and presence of diffuse or localized peritonitis, the treatment vary from primary closure with or without protective colostomy to exteriorization and the Hartmann operation.[9] In our case there was minimal contamination so primary closure of ileum was done.

The purpose of this report is to point out the potential for serious gastrointestinal damage associated with not only high voltage electrical injury but also with low voltage electrical current. Visceral injuries are rare in electrical burns, but they should be suspected when abdominal symptoms appear. A prompt diagnosis and timely intervention usually result in favourable outcome.

## Footnotes

**Source of Support:** Nil

**Conflict of Interest:** None declared

